# Molecular Mechanisms Between Canine Parvovirus 2 and Host Interactions: From Viruses to Innate Immune Signaling Pathways

**DOI:** 10.3390/v18090985

**Published:** 2026-09-08

**Authors:** Guanyu Zhao, Xinying Yang, Yuehua Li, Muzi Li, Shiping Song, Jinghan Wang, Cong Liu, Zequn Dong, Zhenhua Gong, Junhui Liu

**Affiliations:** 1 Carnivore Disease Detection Laboratory, China Animal Health and Epidemiology Center, Qingdao 266032, China; 2 Institute of Special Animal and Plant Sciences, Chinese Academy of Agricultural Sciences, Changchun 130112, China; 3 College of Veterinary Medicine, Qingdao Agricultural University, Qingdao 266109, China

**Keywords:** canine parvovirus, innate immunity, signaling pathway

## Abstract

Canine parvovirus 2 (CPV-2) is a major pathogen causing acute haemorrhagic enteritis and myocarditis in dogs. Since the discovery in the late 1970s, the original CPV-2 strain has evolved through VP2 mutations into new subtypes CPV-2a, 2b, and 2c. The viruses pose a persistent threat to canine health worldwide. This review summarizes the innate immunity evasion tactics employed by CPV-2, focusing on the interactions of various viral proteins and host signaling pathways. CPV-2 regulates the activation and inhibition of Toll-like receptors (TLRs), thereby influencing the downstream MyD88/TRIF signaling pathway and modulating the activation of NF-κB and interferons. CPV-2 exerts bidirectional regulation of apoptosis, thereby modulating host innate immune responses and promoting viral replication. CPV-2 employs multiple strategies to evade the humoral and adaptive immune systems, including evolutionary escape through rapid replication and the accumulation of mutations. Clarifying the key mechanisms underlying the defective TLR activation, the immunomodulatory functions of CPV proteins, and cross-species transmission of CPV-2 among animals and the potential zoonotic risk to humans can provide a crucial theoretical basis for precise virus control, understanding immune evasion, and assessing zoonotic risks, with significant value for both vaccine development and basic research.

## 1. Introduction

Parvoviruses are ancient viruses. Parvoviruses can infect a wide range of animals, including humans, and infect different tissue types. In recent years, outbreaks of human parvovirus have increased significantly. New strains such as porcine parvovirus type 8 (PPV8) emerged [1]. A novel goose parvovirus (GPV) associated with short-beak dwarf syndrome (SBDS) was also identified [2]. Furthermore, canine parvovirus (CPV-2), a significant animal pathogen, attracts widespread attention. This is due to the virus’s demonstrated potential for cross-species transmission. Such transmission makes CPV-2 prevention and control more complex and challenging.

The origin of CPV-2 can be traced back to the late 1970s. At that time, a novel pathogen emerged that caused acute haemorrhagic enteritis and myocarditis in dogs. The new pathogen was subsequently named CPV-2 to distinguish the newly identified pathogen from another parvovirus prevalent at the time—canine minute virus (CnMV). CnMV, previously designated as canine parvovirus type 1 (CPV-1), is associated with neonatal mortality in dogs. Currently, CnMV belongs to the genus Bocavirus, together with bovine parvovirus (BPV) and human bocavirus (HBoV) [3]. Genetically and antigenically, CnMV is unrelated to CPV-2. Research indicated that CPV-2 continued to evolve during transmission, and there were reports that CPV-2 was successfully spread to pigs [4]. In 1979 and 1980, monoclonal antibodies were used to identify a novel strain of canine parvovirus type 2, which was named CPV-2a. During the 1980s, the virus underwent a further antigenic drift, and the new variant was designated as Canine Parvovirus type 2b (CPV-2b) [5]. In 2000, a new mutation was first identified in Italy, giving rise to another antigenic variant, called CPV-2c [6]. These variant viruses coexisted with CPV-2 and continued to circulate.

Canine health is regarded as a major concern. CPV-2 poses serious threats to canine health due to the virus’s long-term survival in the environment and high transmissibility. Particularly in unvaccinated puppies, the mortality rate can reach as high as 91% [7]. CPV has the ability to survive in the environment for a long time, thereby representing a continuous threat to canine health. This in turn adversely affects the development of the dog breeding and pet industries and causes economic losses. This paper reviews the research progress on CPV pathogenesis, focusing on the pathogenic characteristics, loci of action, and pathogenic mechanisms leading to immune response malfunction.

## 2. Biological Characteristics and Host Range Determinants of CPV-2

Canine parvoviral enteritis is recognized as a highly contagious disease caused by CPV-2. CPV-2 belongs to the family *Parvoviridae*, subfamily *Parvovirinae*, and genus *Protoparvovirus* [8]. CPV-2 is classified in the genus *Protoparvovirus* and is a member of the species *Carnivore protoparvovirus 1*, along with Feline panleukopenia virus (FPV), Mink enteritis virus (MEV), and Raccoon parvovirus (RPV) [9]. CPV-2 has been characterized as a non-enveloped virus with an icosahedral capsid. The viral genome consists of a linear, single-stranded DNA molecule approximately 5 kb in length [10].

The CPV-2 genome contains two open reading frames (ORFs). One encodes two non-structural proteins: non-structural protein 1 (NS1) and non-structural protein 2 (NS2). The other encodes two structural proteins: viral protein 1 (VP1) and viral protein 2 (VP2) [11]. Among these, VP1 and VP2 are produced through alternative splicing of mRNA, and VP2 accounts for approximately 90% of the viral capsid [12]. Additionally, there is another protein, VP3. VP3 is the product of the proteolytic processing of VP2 [13]. During the late phase of CPV-2 infection, the P38 promoter drives the transcription of the capsid protein precursor mRNA. This precursor mRNA is then modified by alternative splicing. Subsequently, a leaky scanning mechanism of translation initiation is utilized. The two structural proteins, VP1 and VP2, are synthesized from different start codons. The *VP1* gene is 2181 bp in length and encodes 726 amino acid residues. Approximately 5–6 copies of this protein are present in the viral capsid [14]. VP1 confers the major antigenicity of the virus [15] and is a key target for vaccine design. The *VP2* coding gene is 1755 bp in length and encodes 584 amino acid residues. Compared to the VP1 protein, VP2 lacks 143 amino acid residues at the VP2 N-terminus. VP2 is an important component of the viral capsid, with approximately 54 to 55 copies present in each capsid. VP2 contains the major antigenic determinant cluster and the binding site for the transferrin receptor (TfR) on the host cell membrane [14]. After the viral capsid assembly is complete, approximately 19 amino acid residues at the N-terminus of VP2 are further cleaved off, resulting in the formation of the VP3 protein. VP3 is produced exclusively in intact infectious viral particles. The nonstructural proteins NS1 and NS2 are synthesized by early transcription and translation from the P4 promoter. The *NS1* gene is 2007 bp in length and encodes 668 amino acid residues. The NS1 protein can influence viral replication and trigger apoptosis via a caspase-dependent pathway [16]. The NS1 protein relies on the host cells to access the cellular replication and transcription machinery. The NS1 protein also depends on two DNA-binding domains to regulate viral genome replication and activate the P38 promoter [17]. The *NS2* gene is 531 bp in length and encodes 176 amino acid residues. The NS2 protein can promote viral release and also can provide auxiliary support for NS1 function (Table 1).

CPV-2 can generate mutant strains through antigenic drift, genetic recombination, and other mechanisms [18,19]. Currently, the virus is mainly divided into four subtypes: CPV-2, CPV-2a, CPV-2b, and CPV-2c [20]. Among these, CPV-2 is the original strain, originating from mutations at specific amino acid sites within FPV [21]. Subsequently, the CPV-2a, CPV-2b, and CPV-2c variants gradually appeared [22]. Specific mutations within the VP2 capsid protein are exhibited by each [23,24]. Compared with FPV, the corresponding VP2 amino acid changes in CPV-2 are Arg80Lys, Lys93Asn, Val103Ala, Asp323Asn, Asn564Ser, and Ala568Gly [25]. These changes enable CPV-2 to acquire the ability to bind to the canine TfR, enabling infection of canids. However, infectivity for felids is simultaneously lost [26]. Although CPV-2 is a DNA virus, the genomic substitution rate of CPV-2 is similar to that of RNA viruses [27]. Compared to CPV-2, CPV-2a has five amino acid mutations on VP2: Met87Leu, Ile101Thr, Ala300Gly, Asp305Tyr, and Asn375Asp. CPV-2a became the prevalent strain after 1979 [8,28] and regained the ability to infect cats and other carnivores [29]. CPV-2b is characterized by the Ser297Ala mutation [30]. The host range of CPV-2b largely overlaps that of CPV-2a. However, CPV-2b has a higher prevalence among wild carnivores and wild canids [31]. Compared to CPV-2b, CPV-2c has an Asp426Glu mutation [32]. CPV-2c exhibits greater pathogenicity toward hosts and could infect immunized dogs [8,28]. Furthermore, CPV-2c played a key role in the transmission of the virus from carnivores to artiodactyls and pangolins [33]. The TfR sequence homology among different CPV-2 hosts ranges from 77.2% to 99.0%. The amino acid residues involved in CPV-2 binding are highly conserved. Therefore, CPV-2 may have the potential to infect atypical animal hosts and transmit to humans [33]. However, the current evidence is limited to viral genome identification and in silico prediction, and serological surveillance or direct evidence of infection in human populations is still lacking; therefore, the zoonotic threat of CPV-2 to humans remains unconfirmed. The core distinction among CPV-2a (426Asn), CPV-2b (426Asp), and CPV-2c (426Glu) lies in the mutation at residue 426 [34]. This site is critical for distinguishing subtypes and is located within the antigenic epitope region of the viral capsid. The 426 site could directly affect the efficiency of virus binding to the TfR, and thus enhance the virus’s affinity for canine intestinal epithelial cells [35]. The Ser297Ala mutation in VP2 of CPV-2a and CPV-2b serves as the key molecular marker for identifying the new variant of Canine Parvovirus type 2a (new CPV-2a) and new variant of Canine Parvovirus type 2b (new CPV-2b) [36]. Similarly, in the CPV-2c lineage, mutations at other sites in VP2, such as positions 5, 267, 324, and 370, are the key characteristics for identifying the new variant of Canine Parvovirus type 2c (new CPV-2c) [37,38] (Table 2).

## 3. TLR-Dependent Innate Immune Recognition and Signaling of CPV-2

The innate immune system serves as the host’s first line of defense against viral infection. This defense is mediated by pattern recognition receptors (PRRs) on immune cells—including macrophages, dendritic cells (DCs), and natural killer (NK) cells. The major PRRs involved are Toll-like receptors (TLRs), AIM2-like receptors (ALRs), DNA sensors, NOD-like receptors (NLRs), and RIG-I-like receptors. These receptors recognize pathogen-associated molecular patterns (PAMPs) derived from pathogens. Among these PRRs, TLRs can initiate acute immune responses and induce, coordinate, and regulate the quality of adaptive immune responses [39].

Based on the amino acid sequences and genomic structures, TLRs can be divided into five subfamilies: TLR2, TLR3, TLR4, TLR5, and TLR9. The TLR2 subfamily includes TLR1, TLR2, TLR6, and TLR10. The TLR3, TLR4, and TLR5 subfamilies are relatively independent. The TLR3 subfamily consists of TLR3. The TLR4 subfamily consists of TLR4. The TLR5 subfamily consists of TLR5. The TLR9 subfamily includes TLR7, TLR8, and TLR9 [40]. The types and numbers of TLRs vary among different animals. Mammals have a total of 13 TLR types, but only 10 have been identified in humans: TLR1 through TLR10. TLR11, TLR12, and TLR13 are unique to mice, and the functions of TLR10, TLR12, and TLR13 are not yet clearly understood [41,42]. The cellular localization and reactivity of different TLRs also vary. Among them, TLR1, TLR2, TLR4, TLR5, TLR6, and TLR11 are expressed in the cell membrane. In contrast, TLR3, TLR7, TLR8, and TLR9 are expressed in intracellular compartments, such as endosomes and the endoplasmic reticulum. Cell-surface TLRs are involved in detecting viral proteins, while intracellular TLRs are involved in detecting viral RNA or DNA [43].

TLRs comprise a conserved N-terminal ectodomain of leucine-rich repeats, a single transmembrane region, and a cytoplasmic Toll/interleukin (IL)-1 receptor (TIR) domain [44]. To mediate these signals, TIR domain-containing adapter molecules—such as Myeloid Differentiation Primary Response 88 (MyD88), TIR-domain-containing adapter-inducing interferon-β (TRIF), TIR-domain-containing adapter protein (TIRAP), and TRIF-related adapter molecule (TRAM)—are recruited to the TIR domains of different Toll-like receptors. When a ligand binds to a TLR, the TLR forms a homodimer or heterodimer. This dimerization then recruits a specific TIR domain-containing adaptor protein and activates downstream signaling. TLRs transmit signals through two main pathways. The first is the MyD88-dependent pathway. Upon recruitment, MyD88 further activates interleukin-1 receptor-associated kinases (IRAKs), which subsequently mediate Toll-like receptor signaling and ultimately lead to the activation of nuclear factor kappa-B (NF-κB), mitogen-activated protein kinases (MAPKs), and activator protein 1 (AP1). The second pathway is MyD88-independent. This pathway uses TRIF as an adaptor and finally activates interferon regulatory factor 3 (IRF3). Activation of IRF3 leads to the production of interferon alpha (IFN-α), interferon beta (IFN-β), and other interferon-stimulated genes (ISGs) [39]. The MyD88 adaptor protein is involved in all TLR signaling pathways except the TLR3 pathway [43]. Both TLR3 and TLR4 can activate the TRIF pathway, ultimately leading to the activation of IRF3. The TLR4 signaling pathway can activate both the MyD88 and TRIF signaling pathways, achieving dual-pathway-mediated signal transduction [44].

CPV-2 infection was associated with changes in the expression of different PRRs, including TLRs, which are known to mediate the production of cytokines and interferons. In particular, during the early stage of CPV-2 infection, changes in the expression of PRRs, including TLRs, were observed. These TLR expression changes suggested their potential involvement in the host response to CPV-2 [45], although direct recognition of CPV-2 PAMPs by TLRs has not yet been experimentally demonstrated. The observed TLR upregulation may trigger downstream signaling cascades, leading to the activation of transcription factors, such as NF-κB and interferon regulatory factors (IRFs), and subsequently inducing inflammation and the adaptive immune response of B and T lymphocytes. In addition to the aforementioned TLR-related expression changes, the viral components of CPV-2 themselves were also confirmed to possess immunomodulatory activity. The non-structural protein NS1 of the virus alone could cause death in mice 4T1 tumor cells. Similarly, polyinosinic:polycytidylic acid (poly(I:C)) alone could also cause death in these cells. However, if the two were administered in combination, they could robustly activate the immune system. Further studies confirmed that the TLR3 ligand poly(I:C) could stimulate the production of inflammatory cytokines and type I interferons (IFNs), and could further enhance type I interferon production through the intracellular receptor melanoma differentiation-associated protein 5 (MDA-5) [46]. These interferons played a crucial role in antiviral defense and immune regulation. Meanwhile, CPV-2 infection was associated with the activation of the Interleukin-17 (IL-17) and Tumor Necrosis Factor (TNF) signaling pathways, both of which played critical roles in the host’s defense against viral attack.

**Figure 1:**
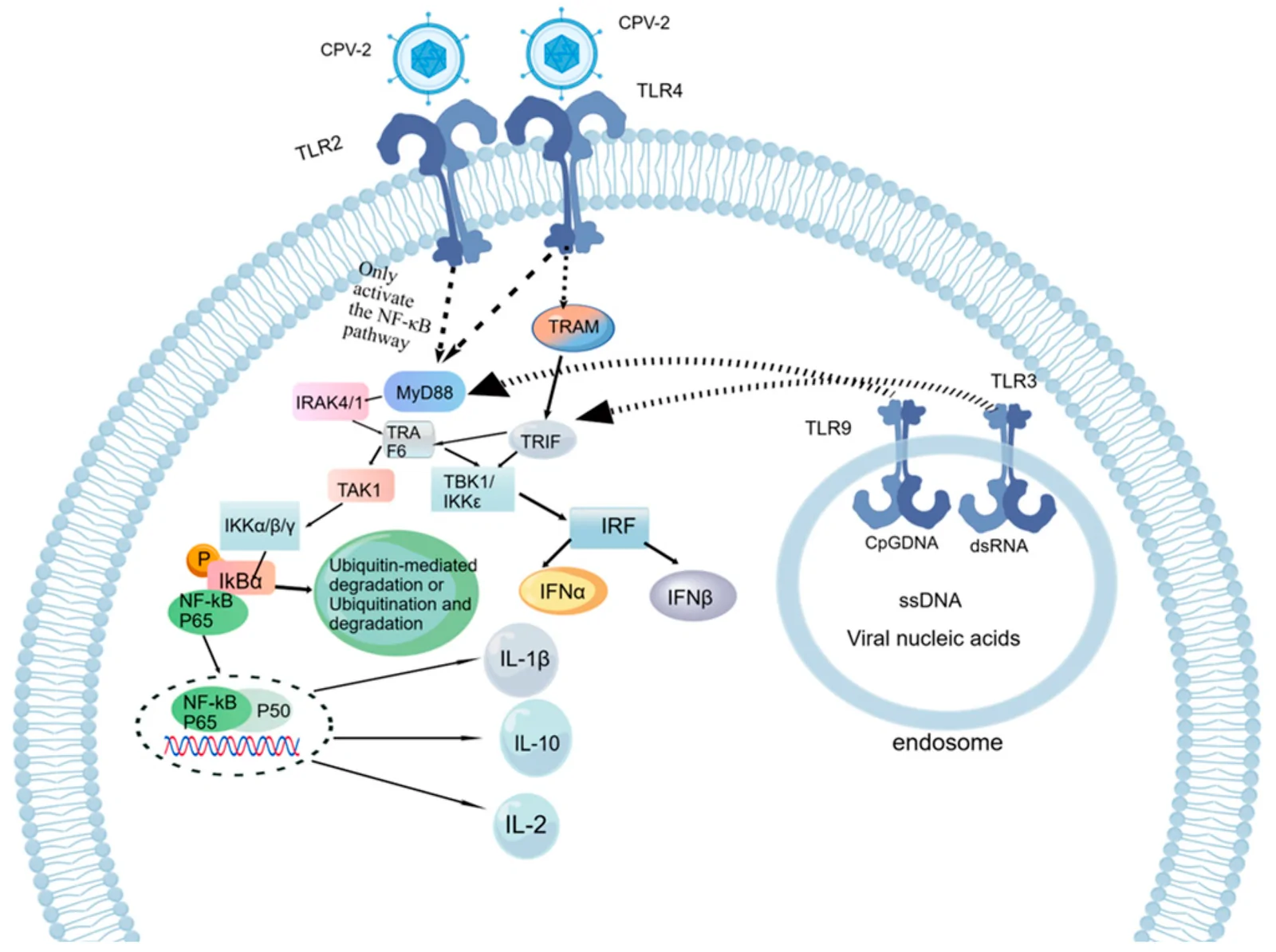
Putative TLR signaling pathways linked to host immune response during CPV-2 infection.

TLRs are PRRs that recognize pathogen-associated molecular patterns and subsequently induce pro-inflammatory cytokines and chemokines, providing a link between innate and adaptive immunity [43,47,48]. Although changes in TLR expression have been reported during CPV-2 infection [45], direct evidence for CPV-2 being recognized by specific TLRs is still lacking. Therefore, understanding the potential role of TLR-related pathways in the host response to CPV-2 infection is crucial for developing new therapeutic strategies, including CPV-2 vaccines adjuvanted with TLR agonists. This understanding is also important for advancing mechanistic research on CPV-2 infection (Figure 1).

## 4. Regulation of Host Innate Responses via CPV-2-Induced Bidirectional Apoptosis

Apoptosis is a genetically regulated process of programmed cell death, serving as an effector mechanism of host defense under the regulation of innate immune signals. This process eliminates virus-infected cells, as well as abnormal cells resulting from DNA damage or developmental processes. The mechanism of apoptosis is complex and involves multiple signaling pathways [49].

After feline kidney F81 cells (F81) were infected with CPV-2, proteomic analysis indicated that CPV-2 was involved in regulating the p53 signaling pathway and apoptosis [50]. In Madin Darby Canine Kidney cells (MDCK), CPV-2 also activated the p53 protein and could induce cell death by regulating factors associated with Bcl-2-associated X protein (Bax) and B-cell lymphoma 2 (Bcl-2) genes [51]. CPV-2 NS1 primarily induced apoptosis through the mitochondrial pathway [16]. The NS1 protein acts on the outer mitochondrial membrane and the DNA damage response process. The NS1 protein activates pro-apoptotic signals such as Bax. The NS1 protein also counteracts anti-apoptotic signals such as Bcl-2, and also triggers oxidative stress [52]. After CPV-2 NS1 induces DNA damage, two apoptotic cascades are simultaneously initiated. First, the phosphorylation and accumulation of H2A histone family member X (H2AX) at the damage sites are promoted, and c-Jun N-terminal kinase (JNK) is activated through a caspase-dependent pathway, leading to chromatin solubilization. Second, PARP is overactivated, and calcium-activated neutral protease 2 (Calpain-2, also known as Capn2) and BH3-interacting domain death agonist (Bid) are cleaved and activated, subsequently leading to the activation of Bax. Truncated apoptosis-inducing factor (tAIF) translocates into the nucleus and interacts with phosphorylated H2AX, ultimately leading to DNA degradation and chromatin disintegration [51,53].

In contrast to the above pro-apoptotic effects, CPV-2 transiently activated the extracellular signal-regulated kinase 1 and 2 (ERK1/2) signaling pathway during early infection. This activation inhibited apoptosis and promoted viral gene expression [52]. The study found that the ERK1/2 signaling pathway exhibited transient activation during the early stage of CPV infection. The intensity ratio of p-ERK1/2 to ERK1/2 increased as early as 15 min post-infection, reached a peak at 30 min, and then gradually declined. By 4 h post-infection, the ratio fell below the mock-infected control group, showing that p-ERK1/2 levels had decreased significantly. Further studies demonstrated that the inhibition of ERK1/2 activation could partially prevent the loss of the mitochondrial membrane potential (ΔΨm) at 2 h post-infection. This finding confirmed that ERK1/2 was indeed effectively activated during the early stage of infection. Activation of ERK1/2 promoted cell survival by inhibiting mitochondrial apoptosis. The ERK1/2 signaling cascade activates cytoplasmic and nuclear substrates, regulating cell survival, division, differentiation, and motility [54]. ERK1/2 signaling regulated cell survival and protected cells from stress caused by viral invasion. By exploiting this signaling mechanism, CPV-2 gained more sufficient time for viral replication [52,54].

In summary, CPV-2 bidirectionally regulated host cell apoptosis through the p53/mitochondrial pathway and the ERK1/2 pathway. The pro-apoptotic signals induced by the NS1 protein facilitated the elimination of infected cells. In contrast, the transient activation of ERK1/2 during early infection inhibited apoptosis and thereby provided a time window for viral replication. This temporal synergy between pro-apoptotic and anti-apoptotic effects constituted an important strategy for CPV-2 to intervene in the host defense effector mechanism regulated by innate immune signaling. This synergy provided new perspectives for understanding the molecular mechanisms underlying CPV-2–host interactions (Table 3).

## 5. Viral Proteins Regulated the Immune Escape Mechanisms of CPV-2

To effectively evade the host immune system, CPV-2 had evolved multifaceted targeted strategies. The virus adopted multiple mechanisms to interfere with host immunity. One of the key targets of these interference strategies is the Toll-like receptor pathway, particularly TLR9. Although TLR9 is a key pattern recognition receptor for recognizing viral DNA, TLR9 mRNA expression is only slightly decreased in CPV-2-infected dogs [45]. This finding indicated that CPV-2 infection mildly downregulated TLR9 expression at the transcriptional level, which might potentially affect the host’s ability to sense viral DNA. However, whether this change represented a specific immune evasion mechanism required further investigation.

Among the non-structural proteins of CPV-2, the NS1 protein plays a crucial role in regulating viral replication and suppressing the host antiviral response. NS1 protein not only blocks the host antiviral response through induction of host cell S-phase arrest [55], but also shuts down host translation by attenuating the phosphorylation of mechanistic target of rapamycin (mTOR) and the downstream effectors eIF4E-binding protein 1 (4E-BP1) and ribosomal protein S6 kinase (S6K), thereby impairing the host immune response and causing pathogenic effects [56]. Given that the NS1 protein exerts immunosuppressive effects through the mTOR signaling pathway, the development of novel vaccines targeting this pathway holds significant potential. In addition to NS1, the other non-structural protein NS2 is also essential for viral replication and host interaction. By constructing multiple CPV-2 NS2 mutants, the study confirmed that mutations and deletions affected the mRNA splicing and protein expression of NS2. This indicated that the degree of influence of NS2 on viral replication depended on the mutation site. Furthermore, the study found that a donor mutant of NS2 led to a decrease in viral infection efficiency [57].

The capsid protein plays a critical role in viral entry and internalization. The unique N-terminal region of CPV-2 VP1 is required for the infectivity of the capsid, but the unique N-terminal region of CPV-2 VP1 is not required for capsid assembly [58]. The N-terminus of VP1 includes a phospholipase A2 (PLA2) domain and a nuclear localization signal (NLS). Activation of PLA2 results in membrane permeabilization, thus promoting CPV-2 release from the endosome. NLS assists the virus in entering the nucleus and enables subsequent nuclear translocation. A study based on the ability of synthetic peptides to mimic the potential NLS of the CPV-2 capsid protein confirmed that a canonical NLS was identified at residues 4 to 13 of the VP1 N-terminus. This nuclear translocation process was also shown to be ATP-dependent [59]. The capsid protein VP2 provides the receptor-binding region. The endocytosis of the virus is mediated through the interaction between VP2 and cellular receptors [59]. Three-dimensional structural analysis revealed that a prominent “spike” structure exists in the threefold axis region of CPV-2 VP2. This structure specifically recognizes host cell surface receptors and mediates the viral endocytosis process, representing a key initial step in viral infection [59]. Five key loop structures are distributed on the surface of the CPV-2 VP2 protein. These loop structures are able to affect the antigenic properties of the virus [30]. The TfR serves as the primary functional receptor on the cell membranes of susceptible animals such as dogs and cats. During CPV-2 infection, specific binding between the VP2 protein and the host cell TfR is achieved. This binding completes the critical steps of viral attachment and entry [60].

In conclusion, the host innate immune response could be cooperatively evaded by CPV-2 through multiple mechanisms. NS1 not only regulates viral replication but also directly suppresses the host immune response. The impact of NS2 on viral replication varied by mutation site, and an NS2 donor mutant reduced viral infection efficiency. VP2 is responsible for mediating the recognition, attachment, and endocytosis of the virus by host cells. Subsequently, VP1 plays a dominant role in the key steps of virus escaping from the endosome and entering the nucleus. These findings revealed the multiple roles of CPV-2 viral proteins in antagonizing host defense, providing a crucial theoretical basis and potential molecular targets for the development of novel antiviral strategies and vaccines targeting virus–host interactions.

## 6. Conclusions

This study summarizes research progress on CPV-2, including etiological characteristics, host immune recognition, apoptosis-mediated regulation of host innate immunity and viral immune evasion strategies.

CPV-2 was identified in 1978. Antigenic variants CPV-2a, CPV-2b, and CPV-2c emerged in 1979. As a DNA virus, the genomic substitution rate of CPV-2 was approximately 10^−4^ substitutions per site per year [27,61]. This is much closer to the substitution rates of RNA viruses (10^−3^ to 10^−5^ subs/site/year) [61]. However, this phenomenon could not be equated with an increase in viral biochemical mutation rate or a decrease in replication fidelity.

The CPV-2–host interaction engages multiple pivotal pathways. VP2 recognizes and binds TfR to facilitate viral cellular entry. The N-terminal PLA_2_ domain of VP1 subsequently induces endosomal membrane permeabilization, while the VP1 NLS mediates nuclear import of the viral genome. NS1 initiates caspase-dependent apoptosis; concurrently, the virus activates ERK1/2 signaling to suppress mitochondrial-mediated apoptosis and sustain viral replication. For immune evasion, NS1 dampens host translation and immune responses via the mTOR pathway. Studies on the pathogenesis and immune evasion of CPV-2 have multiple core contradictions and knowledge gaps. TLR9 mRNA expression was slightly decreased in CPV-2-infected dogs [45]. However, whether this change impaired TLR9 function remained unclear, and the underlying mechanism also remained unclear. Several hypotheses—such as physical masking of viral DNA, direct inhibition of downstream signaling by viral proteins, and host species restriction—remained untested. Molecular epidemiology indicated that CPV-2c had the potential for cross-species transmission. However, conclusive evidence from binding efficiency tests and serological surveys was still lacking. Future efforts are needed to establish a quantitative risk assessment system ranging from receptor binding affinity to human population serosurveys, in order to systematically evaluate the risk of cross-species transmission.

As an important animal pathogen, CPV-2 possesses cross-species transmission capacity and continuously threatens canine health, attracting widespread attention in veterinary and virological research. Upcoming work may explore core nodes of host signaling pathways during CPV-2 infection and resolve the molecular basis for ineffective recognition of the viral genome by innate immune receptors. Related progress facilitates further exploration of CPV-2 pathogenesis and the development of superior preventive and therapeutic approaches.

## Figures and Tables

**Table 1:** Key features of CPV-2 structural and non-structural proteins.

Protein Name	Type	Coding Gene Length (bp)	Amino Acid Residues	Key Functions and Features	Key References
**NS1**	Non-structural	2007	668	Affects replication; triggers apoptosis; regulates genome replication and P38 activation	Gupta et al. (2016) [16]; Niskanen et al. (2013) [17]
**NS2**	Non-structural	531	176	Promotes viral release; assists NS1	Gupta et al. (2016) [16]; Niskanen et al. (2013) [17]
**VP1**	Structural	2181	726	The N-terminal unique region of VP1 contains a PLA_2_ motif for endosomal escape and an NLS for nuclear import.	Allison et al. (2016) [14]; Buonavoglia et al. (2001) [15]
**VP2**	Structural	1755	584	Major capsid component; contains antigenic cluster and TfR binding site	Allison et al. (2016) [14]

**Table 2:** Comparison of the biological characteristics of different subtypes of CPV-2.

Subtype	The Time of Prevalence	Key VP2 Amino Acid Mutations	Major Biological Significance	Key References
**CPV-2**	Prevalent strain after 1978	Arg80Lys, Lys93Asn, Val103Ala, Asp323Asn, Asn564Ser, Ala568Gly	Gained ability to infect Canidae; lost infectivity for Felidae	Doan et al. (2021) [25]
**CPV-2a**	Prevalent strain after 1979	Met87Leu, Ile101Thr, Ala300Gly, Asp305Tyr, Asn375Asp	Regained ability to infect cats and other carnivores	Maganga et al. (2023) [8]; Krishna et al. (2024) [28]
**CPV-2b**	Prevalent strain after 1984	Ser297Ala	Host range largely overlapped with CPV-2a, but had higher prevalence in wild carnivores and wild canids	Li et al. (2022) [30]; Kurucay et al. (2023) [31]
**CPV-2c**	First recorded in 2000	Asp426Glu	Greater pathogenicity than CPV-2; capable of infecting immunized dogs; cross-species transmission.	Maganga et al. (2023) [8]; Krishna et al. (2024) [28]; Zhou et al. (2017) [32]

**Table 3:** Two major biological processes regulated by CPV-2 infection.

Core Mechanism Topic	Key Viral Proteins	Involved Host Signaling Pathways	Key Molecules/Factors	Biological Effect	Key References
**p53 Signaling Pathway and Mitochondrial Pathway Mediate CPV-2-Induced Apoptosis**	CPV-2 NS1	p53 Signaling Pathway, Mitochondrial Intrinsic Apoptosis Pathway, JNK Signaling Pathway	p53, Bax, Bcl-2, H2AX, JNK, PARP, Calpain 2 (Capn2), Bid, tAIF, Caspase	Host cell apoptosis (DNA degradation and complete chromatin decomposition)	Zhao et al. (2016) [50]; Doley et al. (2014) [51]; Arora et al. (2021) [52]
**Mechanism of ERK1/2-Mediated CPV-2 Inhibition of Apoptosis**	CPV-2	Extracellular Signal-Regulated Kinase 1 and 2 (ERK1/2) Signaling Pathway	ERK1/2, p-ERK, mitochondrial membrane potential (ΔΨm)	Inhibit host cell apoptosis, maintain the survival of infected cells, provide sufficient time for viral replication, and ensure the smooth production of progeny viruses	Nykky et al. (2014) [54]

## Data Availability

No new data were created or analyzed in this study. Data sharing is not applicable to this article.
